# Motor fiber organization in the extratemporal trunk of the facial nerve in rats: A retrograde Fluoro-Gold study

**DOI:** 10.3892/etm.2012.701

**Published:** 2012-09-07

**Authors:** LIJIE CHEN, MIN HU, LIHAI ZHANG, SANXIA LIU, JINCHAO LUO, TIANZHENG DENG, YE TAO

**Affiliations:** 1 Departments of Oral and Maxillofacial Surgery and; 2 Orthopaedics, Chinese PLA General Hospital, Beijing 100853;; 3 Department of Stomatology, General Hospital of the Air Force of the Chinese PLA, Beijing 100142, P.R. China

**Keywords:** facial nerve, anatomy, nerve fibers, temporal bone, Fluoro-Gold, rats

## Abstract

Understanding the microanatomy of the facial nerve is vital to functional restoration of facial nerve injury. This study aimed to locate the spatial orientation of five branches in the extratemporal trunk of the rat facial nerve (ETFN). Fifteen adult Sprague-Dawley albino rats were divided randomly into five groups corresponding to the five facial nerves. Fluoro-Gold^™^ (FG) was applied to one branch in all three rats in each group. The trunk of the facial nerve was cut at three points for fluorescence detection. Staining results showed that each branch of the facial motor nerve had a topographical orientation in the distal part of the ETFN. The temporal branch was located in the medial and acroscopic quadrant of the nerve trunk. The zygomatic branch was located in the lateral and acroscopic quadrant. The buccal branch occupied the upper half of the nerve trunk, whereas the mandibular branch occupied the lower half. The cervical branch presented a square-shaped distribution in the lateral nerve trunk. In the middle part of the ETFN, the topographical orientation remained clear, but the FG-labeled zone was extended to some extent. In the stylomastoid foramen region, all branches diffused, thereby blurring the orientation. In conclusion, each branch of the facial motor nerve had a topographical orientation and distribution in the crotch and middle part of the ETFN, but the branches diffused near the stylomastoid foramen.

## Introduction

Knowledge of facial nerve microneuroanatomy is of particular importance in the diagnosis of extratemporal facial nerve lesions, as well as in clinical applications such as fascicular grafting following facial nerve injuries. Animal models are vital for establishing the microneuroanatomy of the facial nerve. The reasons for their frequent use lie in their similarities in gross anatomy and physiology to humans, along with economic advantages and ethical reasons. Thus, they offer an unprecedented opportunity to evaluate the spatial orientation of the extratemporal facial nerve, and to preclinically ascertain the efficacy and safety of newly developed human therapies.

It is generally accepted that the facial motor nucleus has a somatotopic organization (1–5). Whether this is also true for the whole trunk of the facial nerve (WTFN) is a matter of debate, and this has been the subject of numerous investigations utilizing a variety of methods. The use of cadaver dissections (6) is clearly a crude method for examining the organization of axonal populations. In some instances, clinical observations have been combined with neurophysiological stimulation and recording procedures (7). These studies have not convincingly proven the existence of a somatotopic organization. A third way of analyzing the organization of the facial nerve has been to make partial lesions of the facial nerve trunk and to evaluate the resulting functional consequences. This method has also led to different conclusions (8,9). Other methods including radio frequency lesions, crush injuries and various observations have met with varying degrees of success.

Following the application of horseradish peroxidase (HRP) as a neuroanatomical tracing method (10), the question of whether the intrinsic organization of the extratemporal tunk of the facial nerve (ETFN) is topographically or diffusely organized remains to be clarified (11–13).

Fluoro-Gold^™^ (FG) is a fluorescent tracer that has been used successfully in numerous animal models (14–17). Therefore, it was used in this study as a tracer to examine the organization of the ETFN. We aimed to locate the spatial orientation of each facial nerve branch in the distal, middle and proximal parts of the ETFN in Sprague-Dawley albino rats, to improve understanding of the mechanism of facial nerve regeneration after injury of the ETFN.

## Materials and methods

### Facial nerve anatomy

**Figure 1:**
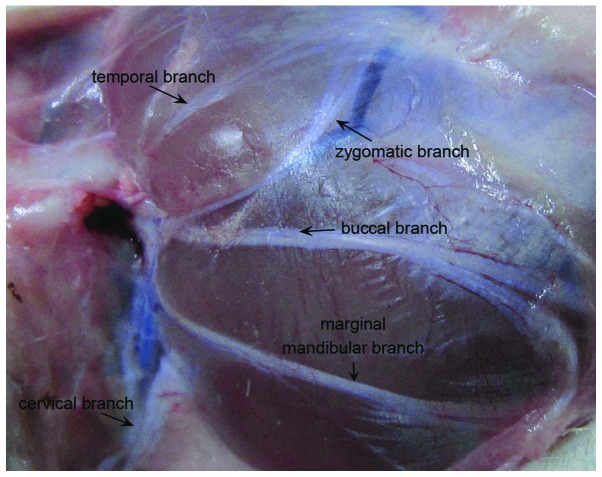
Gross anatomy of the rat face with the extratemporal facial nerve tagged.

The distribution of the facial nerve and the pattern of all the branches of the SD rats appeared similar to those of the human (Fig. 1). The main trunk of the rat facial nerve was divided into five main peripheral branches (temporal, zygomatic, buccal, marginal mandibular, cervical).

### Animals and surgical procedures

**Figure 2:**
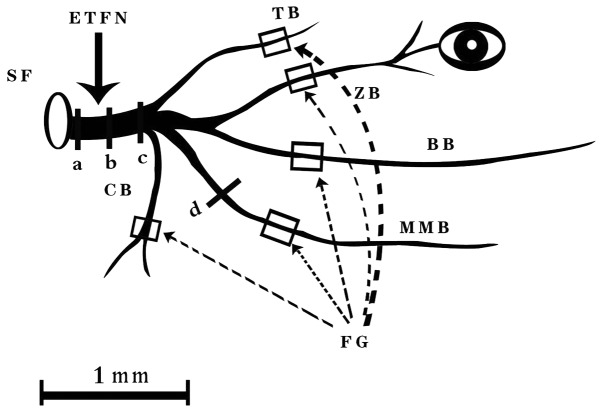
Schema of the extratemporal facial nerve with the location of FG application and detection. (a) Proximal part of ETFN; (b) middle part of ETFN; (c) distal part of ETFN; (d) proximal part of the injection site. TB, temporal branch; ZB, zygomatic branch; BB, buccal branch; MMB, marginal mandibular branch; CB, cervical branch; SF, stylomastoid foramen.

Fifteen adult female Sprague-Dawley albino rats, weighing 250–300 g, were anaesthetized with an intraperitoneal (i.p.) injection of chloral hydrate (300 mg/kg). All animals were kept under standard laboratory conditions (artificial light cycle, 12 h on/off), with tap water and Altromin R/M standard laboratory chow *ad libitum*. FG (Fluorochrome Inc., Denver, CO, USA) was dissolved in distilled water (2% w/v). The animals were randomized to five groups. FG was applied to one branch in each group. The facial nerve branches were dissected carefully under an operating microscope. A 10-μl Hamilton syringe was used to inject 5 μl 2% FG to the proximal end of the nerve under manual pressure. The nerve was clearly transected 10 mm distal to the trunk (Fig. 2). A pipette containing 5 μl FG was then kept in position at the cut end of the trunk for the next 10 min to allow the tracer to penetrate into the tissue. Particular care was taken to achieve complete immersion of the nerve stump in the FG solution. A single 4-0 silk suture (Ethicon) was used to close each wound. The operation was performed bilaterally. The animals recovered from anaesthesia without side effects. After 2 days, they were perfused under deep anaesthesia by thoracotomy and aortic cannulation using 100 ml of 0.1 M phosphate buffered saline, followed by 500 ml of a 4% paraformaldehyde fixative solution, pH 7.4. The facial nerves were dissected from the FG injection site on the face to the stylomastoid foramen immediately after perfusion. The lateral aspect of the nerve was marked by opening the sheath at the crotch of the trunk with a No. 15 Bard-Parker blade. Subsequently, the nerves were cut serially into 10 μm-thick cross-sections on a Leica 1900CM microtome. Care was taken to maintain the serial order of the sections so that the location of the labeled nerve fibers would be apparent. The specimens were examined on three different levels [proximal, medial, and distal parts of the extratemporal trunk of the facial nerve (Fig. 2)], using a Zeiss Axiophot fluorescence microscope and H365 filters (band-pass 365 nm, long pass 397 nm). The study was approved by the PLA Postgraduate Medical School ethics board All animal experiments were carried out in accordance with the guidelines of the Animal Care and Use Committee of PLA Postgraduate Medical School.

## Results

**Figure 3:**
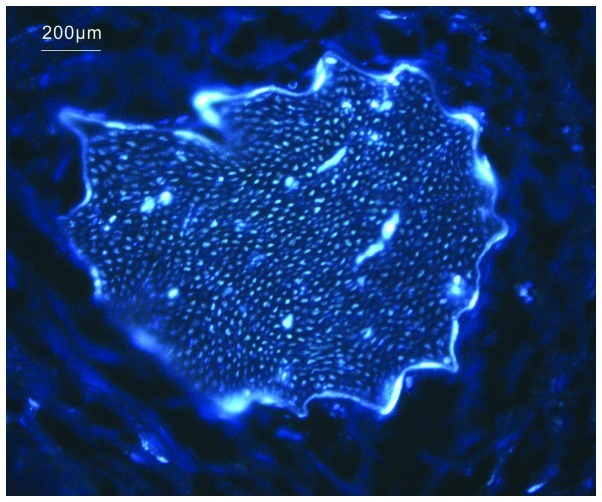
Cross-section of FG-labeled fibers proximal to the injection site.

FG labeled all nerve branches. Bright white dots representing FG-labeled fibers filled the whole cross-section of the branch that was proximal to the injection site (Fig. 3). In general, a definite spatial orientation was retained in the distal part of the ETFN. In the middle part, the FG-labeled zone was partially dispersed, but the orientation was still clear. In the proximal part, however, all branches were diffused with blurred orientation.

### Temporal branch

**Figure 4:**
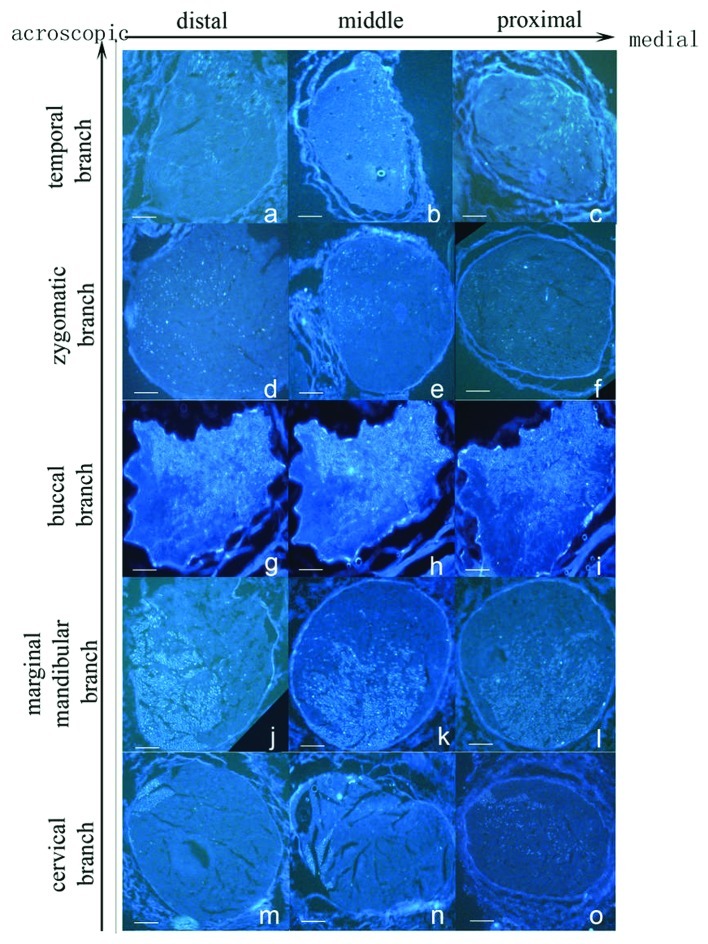
Cross-section of all FG-labeled branches of ETFN. Scale bar, 100 μm.

In the distal part of the ETFN, a crescent-shape labeled zone was found in the medial and acroscopic aspect of the axonal nerve, occupying a quarter of the whole section, with a definite border and a homogeneous distribution (Fig. 4a). The labeled zone extended to the medial part of the ETFN, showing an oval shape (Fig. 4b). The labeled zone extended laterally near the stylomastoid foramen, covering approximately one third of the whole section. In the proximal part of the ETFN, the labeled zone had a circular shape, and the labeled fibers were sparse (Fig. 4c).

### Zygomatic branch

Cross-sections of the FG-labeled zygomatic branch occupied a quarter in the lateral and acroscopic aspect of the nerve in the distal part of the ETFN. The labeled zone presented a ‘c’ shape, and the bright white dots in it were dense in the lateral part (Fig. 4d). The labeled zone expanded inward in the middle part of the ETFN. Most of the bright white dots were concentrated in the lower part of the zone (Fig. 4e). In the proximal part of the ETFN, the labeled fibers were dispersed in the lateral half of the entire section without a definite medial border (Fig. 4f).

### Buccal branch

Labeled fibers of the buccal branch were distributed homogeneously in the upper half of the nerve in the distal part of the ETFN. The labeled zone was much brighter than the temporal and zygomatic branch, with a lower irregular border (Fig. 4g). The labeled area extended inferolaterally slightly into the middle part of the ETFN (Fig. 4h). The bright white dots became sparse in the lower part of the labeled zone in the proximal part of the ETFN. The labeled zone occupied almost three quarters of the whole section with an irregular lower border (Fig. 4i).

### Marginal mandibular branch

When FG was applied to the marginal mandibular branch, an oval zone was found in the inferolateral half area of the nerve, which was slightly smaller than that found in the buccal branch. The labeled zone almost occupied the lower half of the whole section in the distal part of the ETFN (Fig. 4j). In the middle part of the ETFN, the labeled area expanded upward with a moon-shaped unlabeled zone in each lateral border. The upper border of the labeled zone was irregular. The intensity of the labeled fibers in the expanded part was slightly thinner than in the rest (Fig. 4k). The labeled zone dispersed upward and occupied two thirds of the whole section in the proximal part of the ETFN. Labeled fibers in the upper half were sparse compared with those in the lower half (Fig. 4l).

### Cervical branch

Cross-sections of the cervical branch revealed a square zone of axonal labeling in the lateral and acroscopic aspect of the nerve in the distal part of the ETFN, which occupied one fifth of the first quadrant. The border of the labeled zone was definite, which was different from the other four branches (Fig. 4m). In the middle part of the ETFN, the labeled zone stretched out two tapers to the center. The bright white dots distributed homogeneously in the zone (Fig. 4n). In the proximal part of the ETFN, the labeled zone expanded in the center half of the section, presenting an oval zone. Labeled fibers were distributed non-homogeneously in the expanded part and appeared to be fewer than in the square part (Fig. 4o).

The labeled zone of the buccal and marginal mandibular branches were markedly larger than the other three branches in the nerve trunk, with each branch taking over one half of the area. Next were the zygomatic branch, and then the temporal branch. The cervical branch showed the least occupation in the nerve trunk. Nevertheless, among all five branches, the labeled distribution zone of the cervical branch changed most in the distal, middle and proximal part of the ETFN. In the distal part of the ETFN, the labeled zone of all five branches covered the whole cross-section of the nerve trunk, with some areas overlapping. The labeled zone of each branch expanded in the middle part of the nerve trunk, resulting in the corresponding expansion of the overlapping area. In the proximal part of the ETFN, the labeled zone of all five branches continued to expand to one half of the nerve trunk with irregular border. Therefore, it was difficult to distinguish the specific distribution of each branch in this area.

## Discussion

In this study, FG was applied as a tracer in the neuroanatomical tracing method to study the spatial orientation of the ETFN in the rat. Our findings demonstrated that each branch of the facial motor nerve has a topographical orientation in the distal and middle part of the ETFN, but the branches diffuse near the stylomastoid. The question of whether the motor fibers in the WTFN are organized somatotopically or diffusely has been the subject of numerous investigations employing a variety of methods (6–9). However, these methods appear to involve considerable uncertainties. In the early years, neurophysiological stimulation of nerves together with clinical observations was used to detect the topographic orientation of the facial nerve, but the result was only approximate, as the method could only provide information on the rough spatial distribution of nerve branches (7). It is difficult to control the lesion area by cutting the facial nerve, and assessment of the functional consequences is obscured accordingly. Therefore, it is understandable that controversial results were obtained by this method (8,9).

Until now, the neuroanatomical tracing method has been the best way to solve this problem. With this method, axons from different facial nerve branches can be labeled selectively throughout the entire proximodistal length of the WTFN. The neuroanatomical tracing method has promoted the development of neural anatomy. It provides adequate information on the pattern of fiber distribution from different nerve branches. It allows for information to be obtained directly from the nerve, other than by clinical observations or other indirect methods.

With the development of the neuroanatomical tracing method, many commercial products have become available for such studies. HRP was the first and most widely used tracer in the retrograde tracing method. However, it has several disadvantages compared to fluorescent tracers when used in the research of nerve branch orientation in the trunk. HRP requires a series of complicated procedures before developing color. Longer immersion times are required for HRP to reach effective levels of labeling and, therefore, the use of HRP prolongs the anesthesia time and increases the surgical difficulty. Moreover, HRP can label intact, undamaged fibers of passage, thereby interfering with the accurate outcome of the study.

Fluorescent compounds that are used currently as retrograde tracers have several advantages. They can be tested soon after the specimen is obtained without the use of additional staining techniques. This method simplifies the manipulation steps and saves time. Furthermore, it reduces the potential variations resulting from the staining techniques and different experimenters, and, accordingly, improves the accuracy of the study. FG was first introduced in 1986 (18), and since then it has been used frequently as a retrograde tracer in rodents (14–16). It has several advantages, such as complete labeling of the cytoplasm without diffusion, long duration without fading, absorption by damaged fibers only, and easy obtainment (18). FG has been shown to label more neurons than the fluorescent dextrans (19), and it labels more brightly and rapidly than the other tracers (17). FG produces bright white fluorescence under an ultraviolet filter, which is commonly found in fluorescence microscopes.

A number of studies have focused on the spatial relations of the peripheral branches of the intratemporal portion of the facial nerve (ITFN) (9,12,13). The view that facial nerve fibers are organized diffusely has been supported by certain studies utilizing the HRP neuroanatomical tracing method (12,13). Nevertheless, the spatial relation of the ETFN remains unclear. Several preliminary studies have been published in this field. Crumley (11) reported preliminary findings on the fiber organization of the zygomatic branch, but other branches were not included in this study. Choi and Raisman (8) combined hemisection with the neuroanatomical tracing method, and found that 88% of the fibers that supply the temporal branch of the facial nerve travel in the upper half of the facial nerve trunk. However, there have been no studies on the spatial orientation of all branches of the ETFN to date. Therefore, this study was designed to visualize the microanatomy of EFTN and to lay the groundwork for future repair studies.

By dissecting the ITFN, May (9) found that the peripheral fibers in the cat rotate as they travel from the stylomastoid foramen toward the face. However, the result of this study showed that each branch of the facial motor nerve had a topographical orientation in the distal and middle part of the ETFN, but that the branches became diffuse near the stylomastoid foramen.

In general, the results of this study also have implications for clinical practice. Understanding the spatial relations of the facial nerve fibers enables better understanding of the mechanisms of certain diseases, such as Bell's palsy, a unilateral paralysis of the peripheral facial nerve. Our study demonstrates that the branches become diffuse near the stylomastoid foramen. It also explains why suturing complete lesions of the ITFN by intrafascicular repair provides little additional positive effect on the recovery of the nerve function by trying to match the ITFN fibers in the proximal and distal stumps. However, the results of this study also showed that each branch of the facial motor nerve had a topographical orientation in the distal and middle part of the ETFN. Thus, they indicated that intrafascicular suturing may provide a positive effect on functional recovery if the injury is located between the distal and middle part of the ETFN. It can also be concluded that as the injury comes near the stylomastoid foramen, the functional recovery is poorer. The microanatomy of the facial nerve is an important basis of the mechanism of facial nerve regeneration following injury. Understanding the spatial orientation of the ETFN will be beneficial to find new methods of repairing facial nerve injuries. A particular conduit that is designed to bridge the gap between the trunk and branches may be advantageous. Such a conduit should consist of one trunk and multiple branches with its shape imitating that of the facial nerve. The inner structure of the trunk refers to the spatial orientation of each branch. If such a conduit could be constructed, it would be possible to achieve functional recovery.

## Abbreviations:

FG: Fluoro-Gold
ETFN: extratemporal trunk of the facial nerve
HRP: horseradish peroxidase
WTFN: whole trunk of the facial nerve
ITFN: intratemporal portion of the facial nerve
